# Estimates of Minor Ocean Tide Loading Displacement and Its Impact on Continuous GPS Coordinate Time Series

**DOI:** 10.3390/s140305552

**Published:** 2014-03-20

**Authors:** Zhao Li, Weiping Jiang, Wenwu Ding, Liansheng Deng, Lifeng Peng

**Affiliations:** 1 School of Geodesy and Geomatics, Wuhan University, 129 Luoyu Road, Wuhan 430079, China; E-Mails: zhao.li@whu.edu.cn (Z.L.); dlseng_2000@whu.edu.cn (L.D.); 2 GNSS Research Center, Wuhan University, 129 Luoyu Road, Wuhan 430079, China; E-Mail: lfpeng@whu.edu.cn; 3 Institute of Geodesy and Geophysics, Chinese Academy of Sciences, 340 XuDong Road, Wuhan 430077, China; E-Mail: dingwenwu@asch.whigg.ac.cn

**Keywords:** ocean tidal loading, minor ocean tides, GAMIT/GLOBK, GPS data reprocessing, weighted root mean square analysis, spectrum analysis, anomalous harmonics

## Abstract

The site displacement due to ocean tidal loading is regarded as one of the largest uncertainties in precise geodetic positioning measurements, among which the effect of minor ocean tides (MOT), except for the 11 main tidal constituents, are sometimes neglected in routine precise global positioning system (GPS) data processing. We find that MOT can cause large vertical loading displacements with peak-to-peak variations reaching more than 8 mm at coastal/island stations. The impact of MOT on the 24-hour GPS solution is slightly larger than the magnitude of MOT loading itself, with peak-to-peak displacement variation at about 10 mm for the horizontal and 30 mm for the vertical components. We also find that the vertical velocity of all the selected stations in the Southwest Pacific was reduced by more than 10% after considering the MOT effect, while stations with weighted root mean square reduced data account for 62%, 59%, and 36% for the up, east, and north components respectively, in particular for most coastal/island stations. Furthermore, MOT correction could significantly reduce the annual signal of the global stacked east component, the near fortnightly and the long-term periodic signals in the up component. The power of some anomalous harmonics of 1.04 cycle per year is also decreased to some extent. These results further proved the benefits of MOT correction in precise GPS data processing.

## 1. Introduction

Time variable deformation of the Earth caused by ocean tides could reach up to 100 mm at some special coast regions [1,2]. With the growing demands for high precision geodetic observations, ocean tidal loading (OTL) correction has come a must in precise global positioning system (GPS) data processing with baseline lengths of up to several thousand kilometers. Up to now, many ocean tide models (OTMs) were provided by the ocean loading service [3], and geodetic users could easily implement OTL corrections by introducing global grid or station list files for different OTMs. The accuracy of the OTL values depend on the errors in the OTM, Green's function, coastline representation as well as the numerical scheme of the loading computation itself. Currently, the largest contributor to the uncertainty of the loading value is the errors of the OTM itself. Therefore, it is recommended to use the most recent OTMs, for example, the TPXO7.2 and the Finite Element Solution 2004 (FES2004) models [4].

The OTL at a particular location on the Earth caused by a given tidal harmonic is computed by integrating the tide height with Green's function over the whole ocean area [5], and the total impact of OTL equals to the effects of all tidal harmonics. The 11 discrete ocean tide constituents are considered sometimes in OTL modeling during routine precise GPS data processing, including displacement induced by four semidiurnal tide waves M2, S2, N2, K2, 4 diurnal waves K1, O1, P1, Q1, together with three long-period waves Mf, Mm, and Ssa [6–8]. Aside from these 11 main tidal components, however, other principle tidal constituents could also cause surface displacements, e.g., the larger and smaller solar elliptic semidiurnal waves T2 and L2, the smaller and small lunar elliptic diurnal waves M1 and J1, the long-period waves Sa Msf, *etc.* [9,10]. The shallow water ocean loading tides (the third-diurnal and higher-frequency) can be large and significant too, for example, the shallow water constituent M4 exceeds 50 cm at several locations on the northwest European shelf, while the amplitude of the shallow water tides S3, S4 and S5 at the Japanese east coast reaches a few millimeters [11,12].

Since early February 2006, Agnew has posted a fortran routine hardisp.f by considering a total of 141 constituent tides from the 11 main tides, which then became a conventional implementation in November to calculate local site displacement due to OTL, and Hugentobler found that completely neglecting the other ocean tides and nodal modulations with only the 11 main tides may lead to errors of up to 5 mm weighted root mean square (WRMS) at high latitudes using the GOT00.2 ocean model [13,14]. Later on, the 141 constituent tides was included in most of the processing of most, if not all, analysis centers (ACs) for the first IGS reprocessing that contribute to ITRF2008 [15,16].

Thanks to the efforts of many OTL researchers, hardisp.f is being continuously updated online. Until now, it includes a total of 342 constituent tides whose amplitudes and phases are found by spline interpolation of the tidal admittances based on the 11 main tides, and has been implemented in most of the ACs' analysis strategy [17,18]. Currently, among the three most widely used precise GNSS data processing software, Bernese has already implemented the 342 constituents OTL correction, GIPSY considered an additional 32 smaller tidal components aside from the 11 principle tides, while the latest version of GAMIT still applied the 11 constituents OTL correction [19–21]. Here, among the total 342 constituents, we define all the other tidal constituents except for the 11 main tides as the minor ocean tides (MOT). Since many routine precise GPS data processing are being done by GAMIT, which is provided free for non-commercial application, the questions raised are what's the magnitude of station's displacement caused by MOT for global IGS stations? Do they have any impact on station's daily positions derived from the 24-hour GPS solution? What's the impact of MOT on the stations' WRMS and long-term velocity? These are the first focus of this paper.

In the spectral domain, it is well known that there is strong correlation between stations' seasonal variation, especially the vertical annual displacement and the surface displacement induced by redistribution of environmental loads [22]. However, recent publications have demonstrated that the imperfect GPS data processing strategy could also produce spurious seasonal signals in the long GPS time series. For example, the unmodeled or mismodeled diurnal and semidiurnal ocean tides could produce spurious signals with periods of nearly fortnightly, semi-annual and annual variations [23–26], while Tregoning and Watson found that neglect of semidiurnal and diurnal atmospheric tides would also introduce anomalous signals with periods that closely match the GPS draconitic annual (∼351.4 days) and semiannual period (∼175.7 days) [27]. These kinds of spurious signals would interfere with the embedded environmental signals, thus resulting in wrong geophysical interpretation of the GPS coordinate time series. King *et al.*, also found that unmodeled subdaily signals would bias low-degree spherical harmonics estimates of geophysical loading at the level of 5%–10% [28]. What's the impact of MOT on the spectrum of global GPS coordinate time series? This is another motivation of this research.

Finally, Ray *et al.*, found that there existed an anomalous harmonic with period as 1.04 cycle per year (cpy) in the stacked global GPS time series, and the possible origin of this anomalous harmonic was from GPS technique errors, e.g., the repeating geometry of the GPS constellation [29]. Whether the coupling between MOT and the 11 main ocean tides would cause these kinds of anomalous harmonics or not is another issue to be resolved.

In this paper, we first determine the magnitude and spatial distribution of global IGS station's displacement caused by MOT. The OTL modeling method including the MOT correction is then implemented in GAMIT by expanding the 11 main ocean tides into 342 constituents. Based on both the original and the modified GAMIT software, the GPS data of 109 globally distributed IGS stations spanning from June, 1998 to December, 2010 has been reprocessed with state of the art models according to IERS Conventions 2010. Finally, quantitative analyses have been done on two sets of GPS coordinate time series in both time and frequency domains to evaluate the contributions of MOT to global GPS coordinate time series. Results of this paper may provide numerical support to the recommended data processing strategy in the IERS Conventions for crustal movement and interpretation of geophysical signal, as well as the target accuracy of ITRF to achieve 1 mm in position and 0.1 mm/a in velocity [30].

## 2. Data Processing

### 2.1. MOT Modeling

In practice, the 3-D site displacement due to OTL is calculated by:

(1) $$\Delta c=\sum_{j}A_{cj}\cos(\chi_{j}(t)-\phi_{cj})$$

where Δ*c* denotes a displacement component (radial, west and south) at a particular site at time *t*, *j* denotes the tidal component set, amplitudes *A_cj_* and phases *ϕ_cj_* describe the loading response for the chosen site. Conventionally, only the impacts of 11 main tides (*j* = 11) are considered in GPS precise positioning (see previous section for details). The astronomical argument *χ_j_*(*t*) for the 11 main tides can be computed with the subroutine ARG2.F provided by IERS Conventions 2010 [31], while the site-dependent amplitudes and phases for these 11 tides can be obtained from the official ocean loading service mentioned in the previous section.

The amplitudes and phases for other tidal component can be calculated from the above 11 main tides by a variety of approximation methods. For instance, if one wishes to correct for the modulating effect of the 18.6-year lunar node, then:

(2) $$\Delta c=\sum_{k=1}^{11}f_{k}A_{ck}\cos(\chi_{k}(t)+u_{k}-\phi_{ck})$$

where *f_k_* and *u_k_* depend on the longitude of the lunar node [4,32]. In more complete methods, the lesser tides are handled by interpolation of the admittances using some full tidal potential development [33], and one of these methods has been chosen as the conventional method to compute the site displacement caused by OTL [4].

Here, we use the conventional routine hardisp.f recommend by IERS Conventions 2010 to model the MOT effects [34]. In hardisp.f, the amplitude and phase of each tidal component of the total 342 ocean tidal constituents is yielded though spline interpolation technique from the 11 main tides, and then the 3-D site displacement caused by the total 342 constituents is calculated from Equation (1) with j equals to 342 [17]. By subtracting the displacement induced by only the 11 main tides, we could obtain the MOT loading time series for a given site. During the calculation, the amplitude and phase of the 11 main tides is derived from the FES2004 model provided by the ocean loading service, and center of mass (CM) correction has been applied.

### 2.2. GPS Data Processing

**Figure 1.**
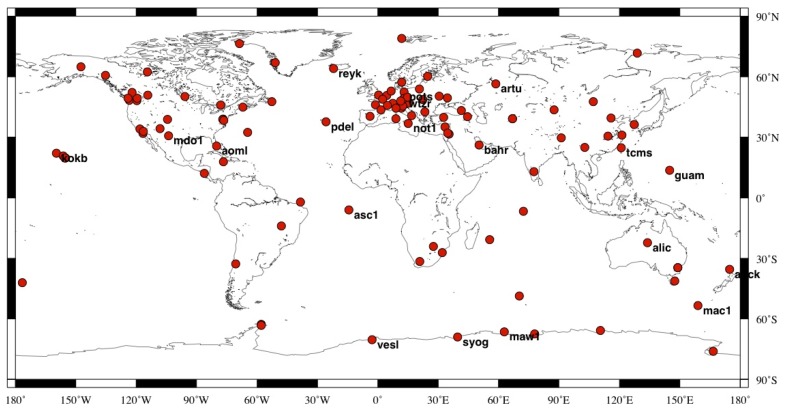
Selected Global IGS reference station distribution. Black texts indicate the name of stations that mentioned in this paper.

To investigate the impact of MOT on the position of global IGS stations, we implement the above recommended conventional OTL correction method in GAMIT 10.4 by substituting the embedded OTL module with hardisp.f. Then a contrast experiment has been designed by reprocessing the GPS data of 109 evenly distributed global IGS stations using the original (Experiment A) and improved GAMIT (Experiment B) [21]. The difference between experiment A and B only comes from the OTL modeling. In experiment A, the OTL correction considered the effects of 11 main ocean tides, while in experiment B, we account for the MOT induced surface displacement by expanding the 11 main tides into 342 ocean tidal constituents. In this way, the contribution of MOT to daily GPS solution and continuous GPS time series of the global IGS stations could be determined by differencing between experiments A and B. Spatial distribution of the selected IGS stations is shown in Figure 1. To reduce time, only data on Wednesdays during the period of June, 1998 to December, 2010 are processed. The global grid of the amplitudes and phases for the 11 main tides with spatial resolution 0.125° × 0.125° is obtained from the FES2004 model [35].

During the data reprocessing, satellite orbits, earth orientation parameters (EOPs), site coordinates together with the tropospheric delay and the horizontal gradient parameters are resolved simultaneously. Loose constraints are implemented on the stations, among which the constraints of IGS core stations are set as 5 cm, and non-core stations are 1 dm. Where possible, ambiguities are fixed to integers [36]. Satellite cut-off elevation angle is chosen as 10 degree, and site-specific, elevation-dependent weighting of the observations based on an assessment of the post phase residuals has been applied [27]. Corrections of solid Earth tide, ocean tide, pole tide as well as atmospheric tide have been implemented [6,37]. Vienna Mapping Function 1 (VMF1) troposphere mapping functions are used to calculate tropospheric delay [38,39]. Where possible, receiver-independent exchange (RINEX) meteorological files (.m) are used to provide pressure and temperature for the a priori zenith hydrostatic delay; otherwise, values from VMF1 are used [40]. Absolute antenna phase center offsets and variations are used (igs08_1636.atx) [41]. Impacts of second and third-order ionospheric delay are considered, during which the International Geomagnetic Reference Field 11 (IGRF 11) is selected to calculate the second-order ionospheric delay [36]. Non-tidal loading corrections are not implemented according to the IERS Conventions 2010 [4]. Finally, gross error rejection and datum transformation are implemented to the daily baseline resolutions using GLOBK to obtain stations' coordinate time series and velocities under the ITRF08 [42]. During the datum transformation, only six parameters, that is three rotation and three translation parameters are estimated to reduce the aliasing effects of the unmodeled surface mass loadings, e.g., the non-tidal atmospheric loading [43].

## 3. Results and Discussion

### 3.1. Loading Displacement Caused by MOT

**Figure 2.**
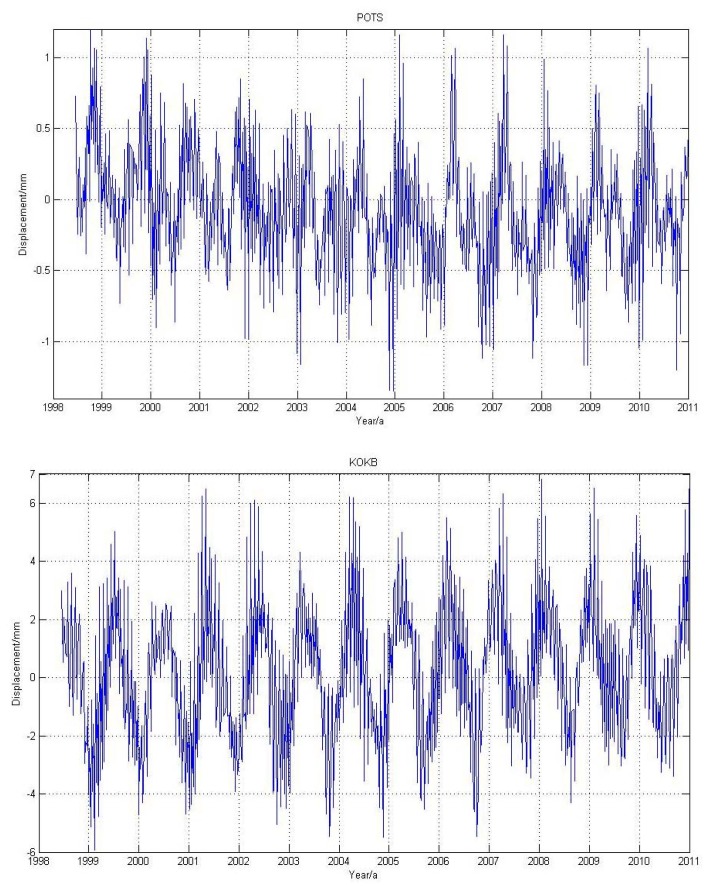
Averaged weekly MOT loading time series for station POTS (**top**) and KOKB (**bottom**).

Using the method outlined in Section 2.1, we obtained the loading time series caused by MOT for the selected 109 stations. Figure 2 shows an example of the averaged weekly MOT height time series for station KOKB (Hawaii, USA) and POTS (Potsdam, Germany). We observe that for inland stations, e.g., POTS, the MOT effect is quite small, with most of the vertical displacement smaller than 0.5 mm. However, for coastal or island stations, such as KOKB, the peak-to-peak vertical displacement caused by MOT reaches 8 mm.

**Figure 3.**
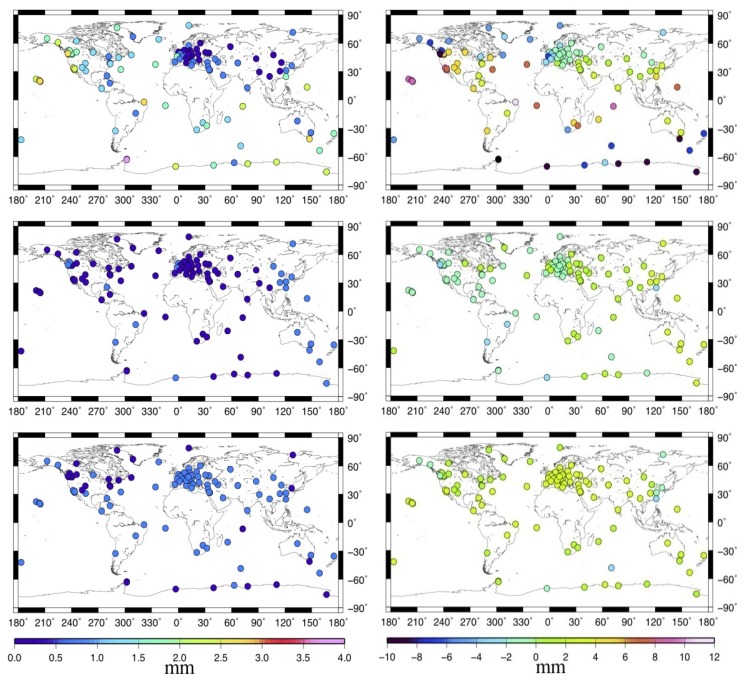
Spatial distribution of the STD (**left**) and the maximum displacement (**right**) of the MOT loading time series. From top to bottom are the U, E, and N components.

To have a better understanding of the global MOT effect, Figure 3 describes the standard deviation (STD, left panels) and the maximum displacement (right panels) of the MOT loading time series for the 109 stations. From top to bottom are the up (U), east (E), and north (N) components. We can see that MOT could only cause small variations in stations' horizontal displacement, with most of the STD and absolute maximum displacement smaller than 1 mm and 3 mm respectively. For the U component, however, it's quite a different story. In general, the vertical displacement induced by MOT becomes smaller with the increasing distance between station and the sea. This is obvious due to the characteristics of the ocean tidal loading itself. Coastal and island stations suffer the most MOT effects, with the biggest STD and the absolute maximum height reaching more than 3.5 mm and 10 mm respectively. For inland stations, the MOT effects are much smaller, with most of the height variation smaller than 1.5 mm, in particular for those in the Eurasia plate, which have almost the same magnitude as that of the E component.

#### 3.2. 24-hour GPS Daily Displacement Caused by MOT

**Figure 4.**
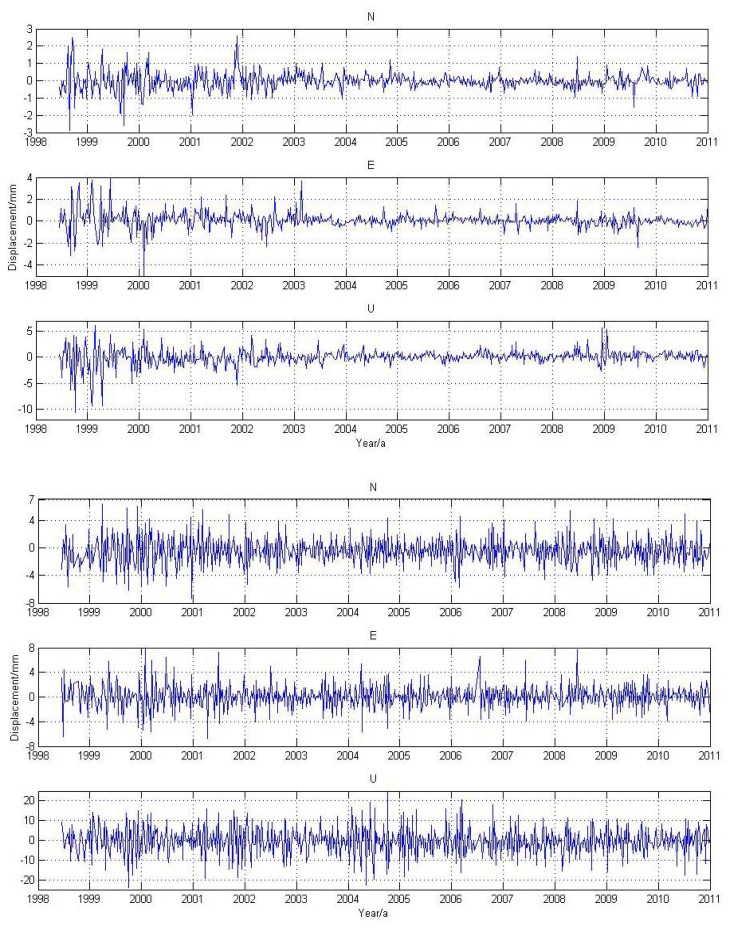
GPS coordinate time series for station ALIC (**top**) and REYK (**bottom**) due to MOT effects.

In Section 3.1, we give the magnitude of stations' displacement caused by MOT. Through reprocessing the GPS data by using the method outlined in Section 2.2, we obtain the MOT effect on the 24-hour daily GPS solutions. Figure 4 shows an example of the weekly GPS time series for station ALIC (Northern Territory, Australia) and REYK (Reykjavik, Iceland) due to MOT effects.

We observe that the magnitude of MOT on the 24-hour GPS solution is slightly larger than that of the MOT loading time series. The peak-to-peak daily displacement caused by MOT for inland station ALIC reaches 5 mm for the N, E component, and 15 mm for the U component (Figure 4, top panels). With respect to the island station REYK, the magnitude is even more than twice as large for both the horizontal and vertical components (see Figure 4, bottom panels).

**Figure 5.**
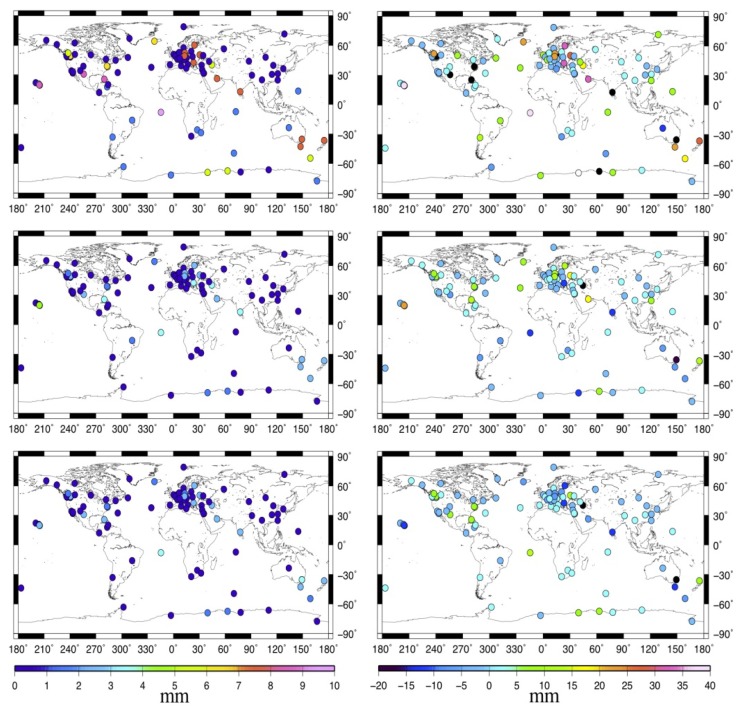
Spatial distribution of the STD (**left**) and the maximum displacement (**right**) of the MOT induced GPS coordinate time series. From top to bottom are the U, E, and N components. Units are in mm. The black and white dots in the figure indicate that the maximum displacement for the station is smaller than the minimum value and larger than the maximum value on the scale.

Figure 5 gives the spatial distribution of the STD and maximum displacement of the MOT induced GPS time series. We observe that MOT exhibits quite a different spatial pattern on the 24-hour GPS solution than that on the pure loading time series. In general, there is no strong correlation between station's MOT induced 3-D displacement and its distance away from the sea.

It influences a lot on many coastal/island stations, for example, station ASC1 (Ascension Island), BAHR (Manama, Bahrain), *etc.* and also some inland station, such as WTZR (Wettzell, Germany), MDO1 (Texas, USA), *etc.* for both the horizontal and vertical components, among which the STD and the absolute maximum of the GPS height reaches more than 5 mm and 20 mm respectively, while the magnitude for the N and E component is slightly smaller. However, for the rest stations, especially those located in North America and Eurasia plates, MOT only has a small impact on the GPS time series, with most of the 3-D STD and absolute maximum displacement smaller than 1 mm and 5 mm. This may due to the interaction between MOT and the other GPS error sources at different location, since our improved GAMIT estimates the MOT displacement together with other effects epoch by epoch, and then solve stations' daily position through least square methods.

### 3.3. MOT Effect on the Long Term Velocity of Global IGS Stations

**Figure 6.**
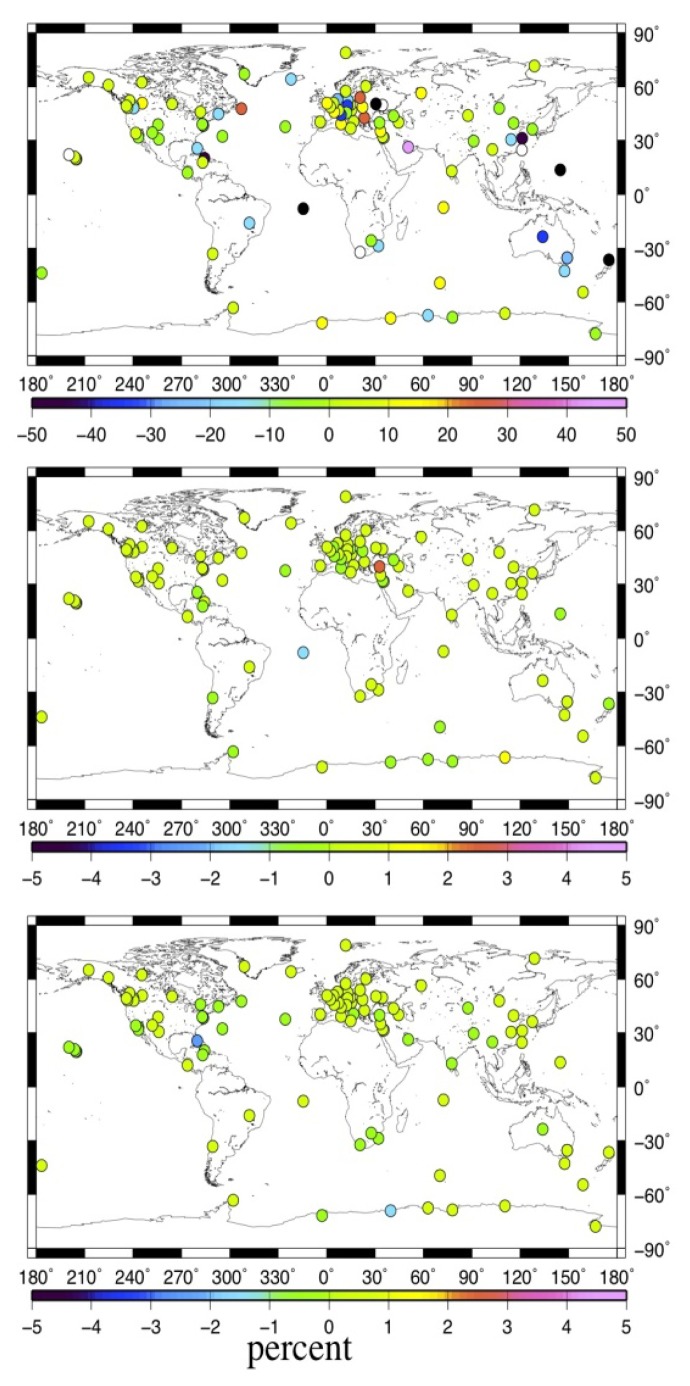
Spatial distribution of the MOT induced long-term velocity variation rate. From top to bottom are the U, E, and N components. Unit of the variation rate is in percentage (%). The black and white dots in the figure indicate that the velocity variation rate for the station is smaller than the minimum and larger than the maximum value on the scale.

Figure 6 shows the spatial distribution of stations' velocity variation rate caused by MOT effect for the U, E and N components.

**Figure 7.**
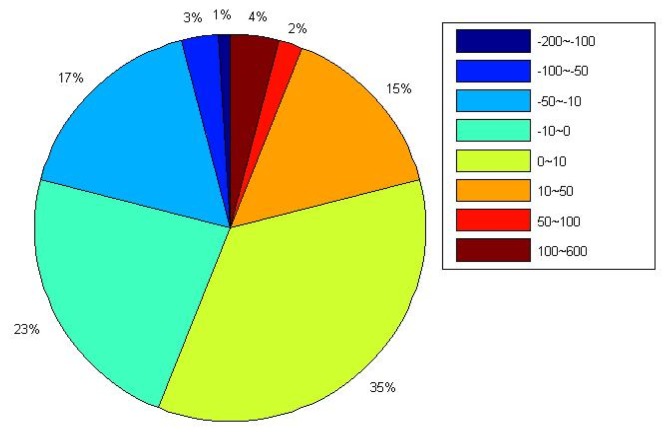
Percentage of stations with MOT induced vertical velocity variation rate inside different ranges. Different color indicates different velocity variation rate range. Unit of variation rate is in percent (%).

Here, we only estimate the velocity of IGS stations with continuous observation span of more than three years, that is 156 GPS weeks, so as to ensure the reliability of the statistics. The basic equation can be written as:

(3) $$\text{vel}\_\text{diff}\%=\frac{vel_{342}-vel_{11}}{vel_{11}}\times 100$$

where *vel_diff* denotes the velocity variation rate for each component, *vel_11_* and *vel_342_* indicate the long-term velocity derived before and after considering the MOT effect respectively. To highlight the impact of MOT on the long-term velocity of the U component, Figure 7 illustrates the percentage of stations with MOT induced vertical velocity variation rate within different ranges.

From Figure 6, we observe that MOT could only cause very small horizontal velocity change, with 98% of the selected stations' velocity variation smaller than 1% for both the E and N components (middle and bottom panels, Figure 6), and even the maximum variation at the island stations are smaller than 3%, e.g., AOML(Florida, USA). Nevertheless, Figure 7 and the top panel of Figure 6 show that the vertical velocity change caused by MOT is quite big, and there are also obvious spatial characteristics, where the variation rate decreased with stations' distance away from the sea. Those with vertical velocity change larger than 10% account for 42% of the selected stations, including most coastal/island stations, among which the maximum variation rate reaches more than 50%, for example, station TCMS (Taiwan, China), GUAM (Dededo, Guam), AUCK (Whangaparaoa Peninsula, New Zealand), *etc.* Another interesting finding is that all the selected stations in the southwest Pacific exhibit a vertical velocity decrease of more than 10% after considering the MOT effect. This may indicate a more precise data processing strategy for GPS stations in this area that applied in the field of sea level study.

Arnadottir *et al.*, found that the vertical motion of IGS reference stations mainly due to effect of post glacial rebound (PGR) [44]. From our results, however, MOT could also result in considerable vertical velocity change, especially for those along the coast or in ocean areas. For example, the vertical velocity of station REYK in Iceland, together with station VESL, SYOG and MAW1 in Antarctica varied by more than 10% after taking MOT into account. Hence, we conclude that MOT may also be a potential candidate for the vertical motion of global IGS stations. Due to the big vertical velocity change caused by MOT for coastal/island stations, in particular for those in the southwest Pacific, we suggest that MOT would be better corrected during high-precision GPS data processing for coastal and ocean regions, so as to obtain more reliable vertical motions for the applications of GPS in geodynamics, such as PGR, sea level studies, *etc.*

### 3.4. WRMS Analysis

**Figure 8.**
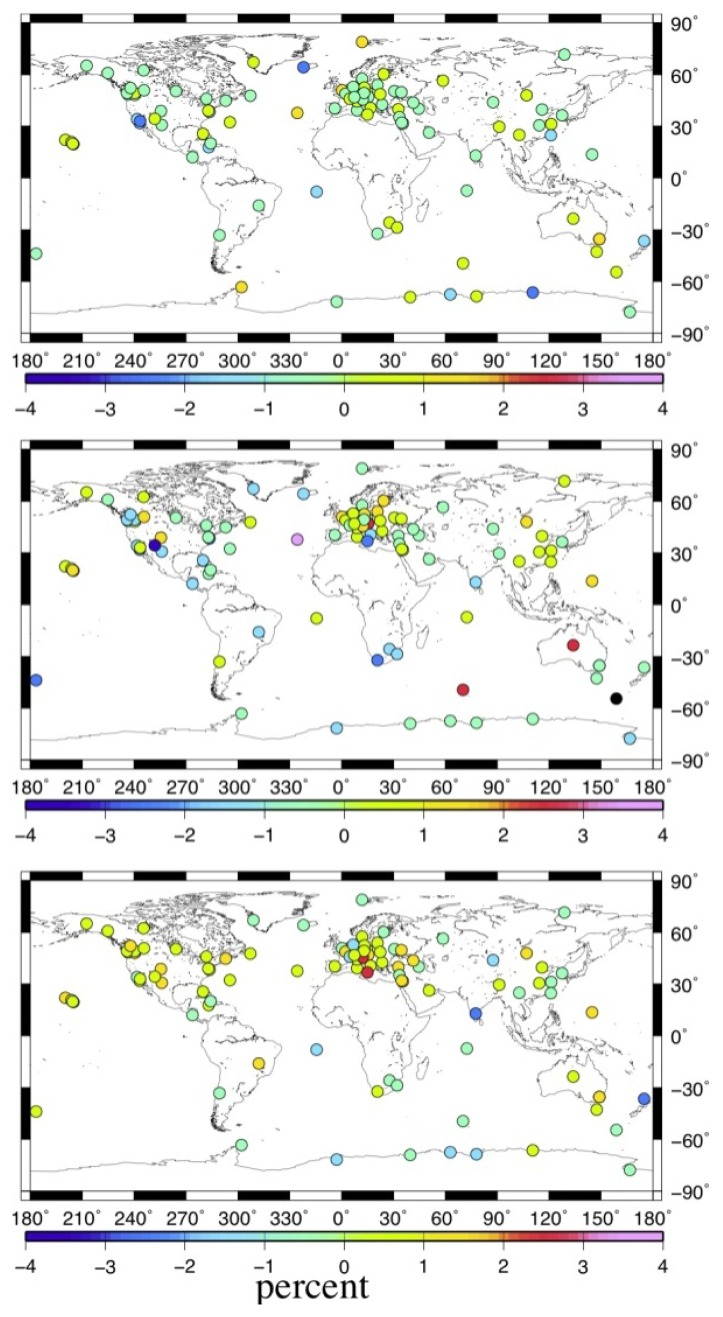
Spatial distribution of the MOT induced WRMS variation rate. Black dot in the figure indicates that the WRMS variation rate for the station is smaller than the minimum value on the scale. From top to bottom are the U, E, and N components. Unit of the variation rate is in percentage (%).

**Figure 9.**
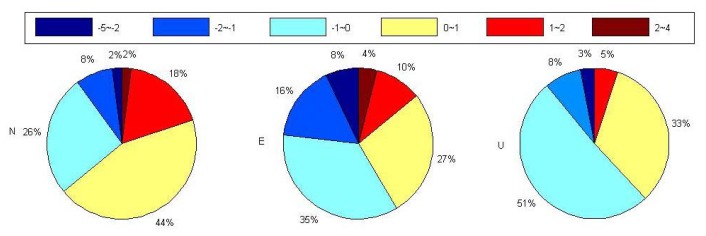
Percentage of stations with the WRMS variation rate inside certain range. Different color indicates different WRMS variation rate in unit of %. (**Left**: N component, **middle**: E component, **right**: U component).

To investigate the impact of MOT on the non-linear characteristics of global IGS stations, we calculate the weighted root mean square (WRMS) of the coordinate time series for the 109 stations before and after considering the MOT effect. Here, we also only estimate the WRMS of IGS stations with continuous observation span of more than three years. Figure 8 illustrates the spatial distribution of the MOT induced WRMS variation rate. Negative means that the WRMS reduced after implementing MOT correction. Figure 9 gives the percentage of stations with the WRMS variation rate inside different ranges. For the definition of the WRMS and its variation rate, please refer to [22] for detail.

From Figures 8 and 9, we observe that MOT could only introduce small WRMS variations of the global IGS stations, with maximum variation rate smaller than 5% at coastal/island stations. Different from the results in previous sections, the vertical component exhibits the smallest impact. Except for only several coastal/island stations, e.g., REYK, whose WRMS reduction reaching 3%, stations with WRMS variation rate smaller than 1% account for 84% of the selected stations. For the horizontal components, the impact of MOT is slightly larger. 59% of stations' WRMS in the E component are reduced after considering the MOT effect, with maximum reduction of 1%∼5% at most coastal and island stations, such as station MAC1 (MacQuarie Island, Southern Ocean). With respect to the N component, however, stations with WRMS reduced account for only 36%, and most of them are located along coasts or on the island, with maximum WRMS reduction smaller than 3%. Nevertheless, implementing MOT correction could also increase the WRMS for some global IGS stations, in particular for the N component. For example, the E component of station PDEL (Ponta Delgada, Portugal) and the N component of station NOT1 (Sicily, Italy) exhibit a WRMS increase of about 3% after considering MOT effect.

Until now, there is still big discrepancy between global IGS stations' non-linear position time series and the environmental loading induced displacement [22,29,45,46]. One of the major factors that caused the discrepancy is known as the GPS related error [29,47,48]. Our MOT modeling here at the observation level during the GPS data processing may indicate a possible source that would narrow the gap. Further work still needs to be done on the contribution of MOT to the inconsistency between GPS coordinate time series and the surface displacement driven by environmental loading.

### 3.5. MOT Effect on the Spectral Characteristics of Global IGS Stations

**Figure 10.**
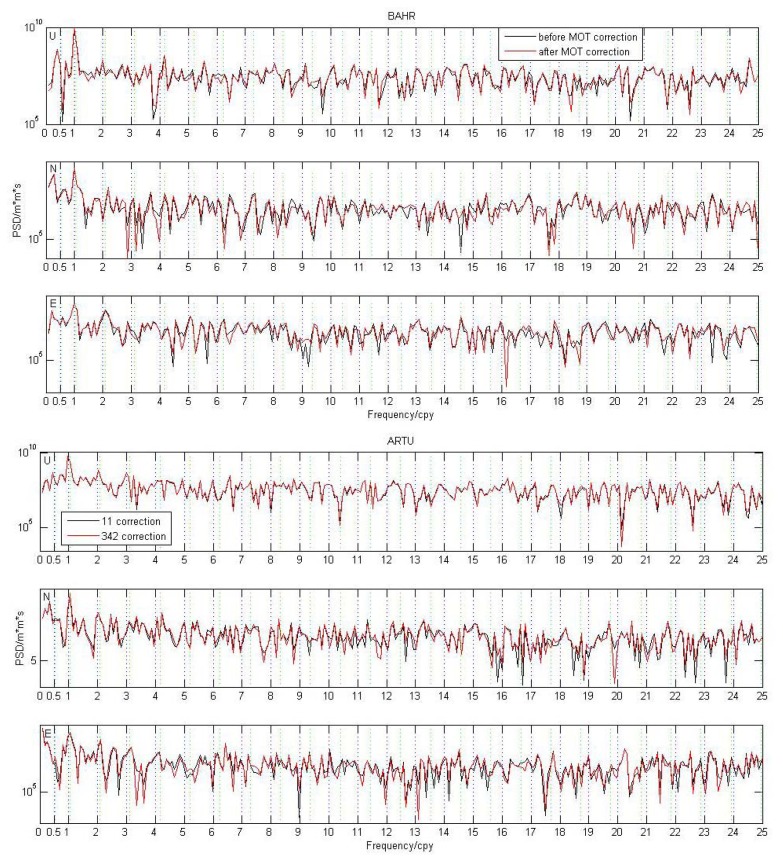
PSD results for the U, N, and E components of station BAHR (top panels) and ARTU (bottom panels) before and after MOT correction. Vertical black and green dash lines represent harmonics of 1 *cpy* and 1.04 *cpy*.

From Figure 2, we observe that the loading displacs some periodic characteristics with period as about two years, in particular for island station KOKB, but the MOT induced 24-hour GPS position time series does not show obvious periodic features (see Figure 4). To better investigate the impact of MOT on the periodic motion of global IGS reference stations, we calculate the power spectrum density (PSD) of the 109 stations in both Experiment A and B through using CATS software [49]. Figure 10 gives an example of the PSD result for station BAHR and ARTU (Arti, Russia). Black and red curves in the figure represent the PSD before and after MOT correction respectively. Vertical black and green dash lines denote the harmonics of 1 *cpy* and 1.04 *cpy* respectively. We observe that MOT could only cause small periodic difference, although the magnitude for coastal station BAHR is slightly bigger than inland station ARTU. We find that strong annual signal exists for each component of the two stations both before and after MOT correction, which also exhibit an increase except the East component of station BAHR, in particular for the N and E components of station ARTU, which increased by 23% and 10% respectively. However, there is no regular rule to describe the characteristics of the other periodic signals caused by MOT.

From Figure 10, we could also see the existence of the anomalous harmonics of 1.04 *cpy* ± 0.008 *cpy*, which is now known as the GPS draconitic year [29], for example, the 4th draconitic signal in the U and N components of station BAHR, the 1st draconitic signal in the N and E components of station ARTU, no matter MOT correction is applied or not. Moreover, the 1st and 4th draconitic signal in the U component of BAHR decreased by about 7% and 55% respectively after considering the impact of MOT, while the 1st draconitic signal in the E component and the 2nd draconitic signal in the N component of station ARTU also decreased by 9% and 18%. Until now, the well-known origin of the above anomalous harmonics comes from the GPS technique error, such as long-period GPS satellite orbit modeling error, the repeating geometry of the satellite constellation with respect to the tracking stations, *etc.* [29]. Our PSD results from these two stations indicate that MOT could also be a possible origin that caused the draconitic signal.

**Figure 11.**
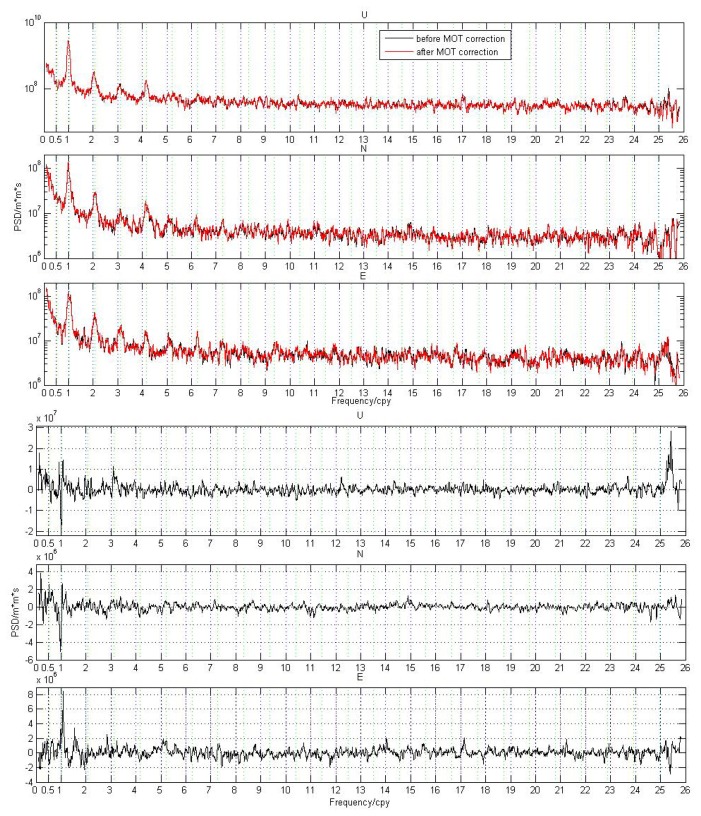
Top: Global stacking PSD results for the filtered U, N, and E component before and after MOT correction. Bottom: PSD difference caused by MOT for each corresponding component. The vertical black and green dash lines in the figure have the same meaning as that in Figure 10.

**Figure 12.**
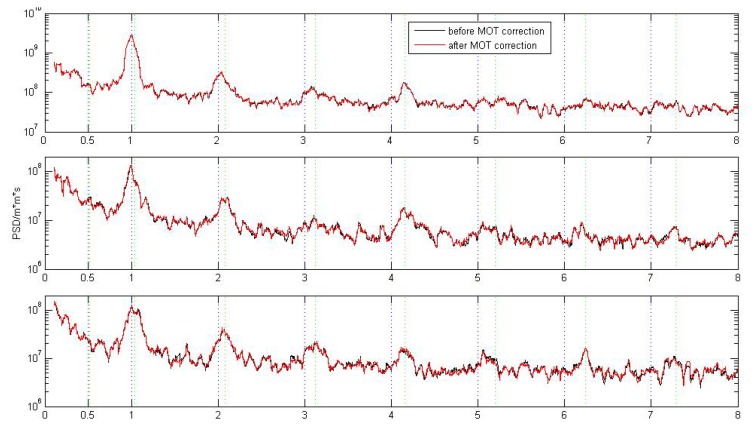
Expanded view of Figure 11 (top panel).

Figure 10 only represents the PSD results for single stations. Since there is no big difference in the PSD characteristics between coastal/island stations and inland stations, here we stack and filter the PSD time series of the selected 109 stations for the N, E, and U components respectively, so as to have a global view of the PSD difference caused by MOT. For the implementation of the PSD stacking and filtering, please refer to [29] for detail. Figure 11 shows the final filtered PSD stacking results for each component before and after MOT correction (top panels), together with their differences (bottom panels). In the bottom panels of Figure 11, positive value means that the PSD amplitude is decreased after correcting MOT effects. To have a more clear view of the dominant power spectra, Figure 12 gives an expanded view of the filtered spectra shown in Figure 11 (top panels) from the 1st to the 7th harmonics of 1.0 *cpy* and 1.04 *cpy*.

From Figures 11 and 12, we observe that no matter MOT correction is applied or not, the annual signal and the anomalous harmonics of 1.04 *cpy* ± 0.008 *cpy* until the 7th draconitic signals are the dominant periodic signals in both the vertical and horizontal components of global IGS stations, and the magnitude in the vertical component is about 10-times larger than the horizontal components. Similar as in Figure 10, the annual amplitude of the E component dramatically decreased after considering the MOT effect, but that of the U and N components exhibit a significant increase. We also find that the amplitude of most abnormal harmonics of 1.04 *cpy* ± 0.008 *cpy* until the 7th draconitic signals decreased to some extent, in particular for the 3rd draconitic harmonic of the U component. This result confirms our previous finding from Figure 10 that MOT may be a possible source that caused the anomalous harmonics of 1.04 *cpy*, although the contribution may be slightly small.

Another interesting result from Figure 11 is that the short-term periodic signals with frequency larger than 25 *cpy*, that is the near fortnightly periodic signal, in the U component show a significant decrease after correcting the MOT effect. This may give some useful clue for us to reduce some of the high frequency periodic signal in the GPS height time series when reprocessing the global GPS data. Furthermore, most long-term periodic signal with frequency equal to or smaller than 0.5 *cpy* in the U and N components also decreased to some degree. This phenomenon confirms the recent finding that the unmodeled diurnal and sub-diurnal periodic signal could indeed propagate and produce spurious long-term signals in the GPS coordinate time series [25]. Except for these mentioned periodic signals, however, the PSD differences that caused by MOT show a random pattern, which is also similar as that shown in Figure 10.

## 4. Conclusions

The effects of MOT are sometimes neglected in routine precise long-baseline GPS data processing. In this paper, we first determine the magnitude and spatial distribution of global IGS station's loading displacement caused by MOT. We find that MOT could only cause small variations in the horizontal loading displacement globally, with most of the STD being smaller than 1 mm. The vertical loading displacement caused by MOT is quite large, and becomes smaller with the increasing distance between station and the sea. This is obviously due to the characteristics of ocean tides. The peak-to-peak MOT induced loading displacement variations reaches more than 8 mm at the coastal/island stations, while most of the height variations for inland station are smaller than 1.5 mm, in particular for those in the Eurasia plate, which have almost the same magnitude as that of the E component.

Secondly, the OTL modeling method including the MOT correction is implemented in GAMIT. Through reprocessing the GPS data of 109 globally distributed IGS stations based on both the original and the modified GAMIT software, we find that the impact of MOT on the 24-hour GPS solution is slightly larger than the magnitude of MOT loading time series itself, and it also exhibits quite a different spatial pattern. There is no strong correlation between station's MOT induced 3-D displacement and its distance away from the sea. It influences a lot on many coastal/island stations (e.g., REYK) and also on some inland stations (e.g., WTZR), among which the STD and the absolute maximum of the GPS height caused by MOT reaching more than 5 mm and 20 mm respectively, with peak-to-peak displacement change at about 10 mm for the horizontal component and 30 mm for the vertical components. For stations located in North America and Eurasia plates, however, MOT only has a small impact on the GPS time series, with most of the 3-D STD smaller than 1 mm. We think that this may due to the interaction between MOT and the other GPS error sources at each epoch and in different places.

We then study the impact of MOT on the long-term velocity of global IGS stations. Our results show that the vertical velocity change caused by MOT is quite big, and also decrease with stations' distance away from the sea. 42% of the selected stations, including most coastal/island stations' vertical velocity change are larger than 10%, and the maximum velocity variation rate reaches more than 50% (e.g., station ASC1, BAHR). Besides, considering the MOT effect could reduce the vertical velocity of all the selected stations in the Southwest Pacific by more than 10%. Thus, we conclude that MOT may also be a potential candidate for the vertical motion of global IGS stations, in particular for those along the coast or in ocean areas, where MOT should be better corrected during high-precision GPS data processing so as to obtain more reliable vertical motion for geodynamic studies, such as PGR, sea level change, *etc.*

Moreover, the impact of MOT on the non-linear characteristics of global IGS stations is discussed. We find that in general MOT would only introduce small WRMS variations in the global IGS stations, even for the coastal/island stations, where the maximum variation rate is smaller than 5%. The U component exhibits the smallest impact, the N component ranks the second, while the E component ranks the third, with stations whose WRMS variation rates smaller than 1% account for 84%, 70%, and 62% of the selected stations. 62%, 59%, and 36% of the stations' WRMS reduced for the U, E and N components respectively after considering the MOT effect, among which the maximum reduction with magnitude of 1%∼5% gathered mostly along coasts or on the island. Nevertheless, implementing MOT correction could also increase the WRMS for some global IGS stations, in particular for the N component (e.g., station NOT1). Further work still needs to be done on whether the MOT modeling at the observation level of GPS data processing would narrow the gap between global IGS stations' non-linear position and the environmental loading induced displacement time series.

Finally, the spectral characteristics of global IGS stations that caused by MOT is analyzed. Our results show that MOT could only cause small periodic difference, although the impact on the coastal stations is slightly bigger than that on the inland stations, and the magnitude of the periodic difference in the vertical component is about 10-times larger than the horizontal components. We find that MOT correction could significantly reduce the annual signal of the global stacked E component, but that of the U and N components exhibit a significant increase, for example, the annual signal of the N component for station ARTU increased by 23%. Furthermore, the near fortnightly periodic signal and the long-term periodic signal with frequency equal to or smaller than 0.5 cpy in the U component show a significant decrease after correcting the MOT effect. This may indicate a more precise data processing strategy for reducing some of the high frequency periodic signals in the GPS height time series, and also support the recent finding that the unmodeled diurnal and sub-diurnal periodic signal would indeed propagate into spurious long-term signals. Finally, we find that MOT could reduce the power of most anomalous harmonics of 1.04 *cpy* ± 0.008 *cpy* until the 7th draconitic signals to some extent, in particular for the 3rd draconitic signal of the U component, thus we conclude that MOT may also be a possible source that caused the anomalous harmonics of 1.04 *cpy*.
